# Supplementary material for: Barriers to effective communication among nurses and family members of patients admitted to the intensive care unit at Muhimbili National Hospital in Dar es Salaam: A descriptive qualitative study
Source: PLoS One. 2025 Sep 4;20(9):e0330374. doi: 10.1371/journal.pone.0330374 (PMC12410765; doi:10.1371/journal.pone.0330374)
Supplement: S1 Text — (DOCX) [file pone.0330374.s001.docx]

**Supplementary File 1: COREQ checklist**

**Filled checklist of Consolidated Criteria for Reporting Qualitative Studies (COREQ)**

| **Item No** | | **Guide Questions/Description** | **Reported on Page #** |  |
| --- | --- | --- | --- | --- |
| **Domain 1: Research team and reflexivity** | | | |  |
| **Personal Characteristics** | | | |  |
| 1. Interviewer/ facilitator | | Which author/s conducted the interview or focus group? | SC conducted the interviews (Pg 6) |  |
| 2. Credentials | | What were the researcher’s credentials? E.g., PhD, MD | SC MSc in Nursing, GL- MSc and MN has PhD (Pg 18) |  |
| 3. Occupation | | What was their occupation at the time of the study? | SC was a student while GL and MN were university lecturers (Pg 18) |  |
| 4. Gender | | Was the researcher male or female? | One female and 2 male researchers (Pg 18) |  |
| 5. Experience and training | | What experience or training did the researcher have? | GL and MN have experience in Research Methodology during their careers as University teachers while SC during his studies and mentorship. |  |
| **Relationship with participants** | | | |  |
| 6. Relationship established | | Was a relationship established prior to study commencement? | No prior relationship existed between researchers and participants. |  |
| 7. Participant knowledge of the interviewer | | What did the participants know about the researcher? e.g. personal goals, reasons for doing the research? | Participants were informed about the purpose of the research and their rights to participate They were ensured of privacy and confidentiality during and after the research (Pg 6,7) |  |
| 8. Interviewer characteristics | | What characteristics were reported about the interviewer/facilitator? e.g. Bias, assumptions, reasons and interests in the research topic | The researchers had a shared interest in the research(Pg 6) |  |
| **Domain 2: study design** | | |  |  |
| **Theoretical framework** | | |  |  |
| 9. Methodological orientation and Theory | What methodological orientation was stated to underpin the study? e.g. grounded theory, discourse analysis, ethnography, phenomenology, content analysis | The framework used was a inductive thematic analysis (Pg 4,6) |  |  |
| **Participant selection** | | |  |  |
| 10. Sampling | How were participants selected? e.g., purposive, convenience, consecutive, snowball | Participants were purposively selected (Pg 5) |  |  |
| 11. Method of approach | How were participants approached? e.g., face-to-face, telephone, mail, email | Participants were contacted physically and face-to-face interviews were conducted (Pg 5,6) |  |  |
| 12. Sample size | How many participants were in the study? | 27 participants took part in the study (Pg 5) |  |  |
| 13. Non-participation Setting | How many people refused to participate or dropped out? Reasons? | Five contacted participants refused to participate due to lack of time, however, of 27 participants who consented to participate none dropped out |  |  |
| 14. Setting of data collection | Where was the data collected? e.g., home, clinic, workplace | The data collection took place at the hospital during clinic visits (Pg 5, 6) |  |  |
| 15. Presence of nonparticipants | Was anyone else present besides the participants and researchers? | No one was present besides the participants and researchers. |  |  |
| 16. Description of sample | What are the important characteristics of the sample? e.g. demographic data, date | Age, sex, occupation, educational background, duration of caregiving, and relationship with the patient (Pg 4) |  |  |
| **Data collection** | | |  | No |
| 17. Interview guide | Were questions, prompts, and guides provided by the authors? Was it pilot tested? | The interview guide was rigorously reviewed and pretested. Follow-up questions/prompts were asked (Pg 5,6) |  |  |
| 18. Repeat interviews | Were repeat interviews carried out? If yes, how many? | No repeat interviews conducted |  |  |
| 19. Audio/visual recording | Did the research use audio or visual recording to collect the data? | The researcher used audio recording to collect data (Pg 6) |  |  |
| 20. Field notes | Were field notes made during and/or after the interview or focus group? | Field notices were taken during the interview(Pg 6) |  |  |
| 21. Duration | What was the duration of the interviews or focus group? | The interviews lasted between 60 to 90 minutes (Pg 6) |  |  |
| 22. Data saturation | Was data saturation discussed? | Data saturation was discussed (Pg 5) |  |  |
| 23. Transcripts returned | Were transcripts returned to participants for comment and/or correction? | No transcripts returned |  |  |
| **Domain 3: analysis and findings** | | |  |  |
| **Data analysis** | | |  |  |
| 24. Number of data coders | How many data coders coded the data? | Three people participated in the coding process (Pg 6) |  |  |
| 25. Description of the coding tree | Did the authors provide a description of the coding tree? | N/A |  |  |
| 26. Derivation of themes | Were themes identified in advance or derived from the data? | Data were inductively analysed (Pg 6) |  |  |
| 27. Software | What software, if applicable, was used to manage the data? | Data were analyzed manually. |  |  |
| 28. Participant checking | Did participants provide feedback on the findings? | Initial findings were shared with a few (Pg 7) |  |  |
| **Reporting** | | |  |  |
| 29. Quotations presented | Were participant quotations presented to illustrate the themes/findings? Was each quotation identified? e.g., participant number | Yes (Pg 10-13) |  |  |
| 30. Data and findings consistent | Was there consistency between the data presented and the findings? | Yes, the authors endeavored to report the findings consistently reflecting on the collected data (Pg 10-13) |  |  |
| 31. Clarity of major themes | Were major themes clearly presented in the findings? | Yes (Pg 10-13) |  |  |
| 32. Clarity of minor themes | Is there a description of diverse cases or a discussion of minor themes? | No |  |  |
