# Supplementary material for: Barriers to effective communication among nurses and family members of patients admitted to the intensive care unit at Muhimbili National Hospital in Dar es Salaam: A descriptive qualitative study
Source: PLoS One. 2025 Sep 4;20(9):e0330374. doi: 10.1371/journal.pone.0330374 (PMC12410765; doi:10.1371/journal.pone.0330374)
Supplement: S2 Text — (DOCX) [file pone.0330374.s002.docx]

## Interview guide (For Family members)

**Title:** ***Barriers to effective communication among nurses and family members of patients admitted to intensive care unit at Muhimbili National Hospital in Dar es Salaam: A descriptive qualitative study***

Date of interview…………………………………..

Interview site………………………...

Interviewee No ………………………

Start time……………………………

End time………………………………

**A: Interviewee background**

Age of participant----------------

Sex ------------------------------------------

Marital status---------------------------------------

Occupation -------------------------------------------

Level of education----------------------------------------

Relationship to the patient --------------------------------------------

Duration of caregiving………………………………………..

**B: Family members’ experience**

1. Please describe your experiences communicating with nurses during ICU admission of your patient
2. Please share with me challenges that you faced when interacting with the nurse
3. How did you navigate through the challenges
4. What are the things that should be done to improve communication interaction

Thank you for your time in this interview; do you have anything else to add? If not, I appreciate it very much!

**END**

## Interview guide (For Nurses)

**Title: *Barriers to effective communication among nurses and family members of patients admitted to intensive care unit at Muhimbili National Hospital in Dar es Salaam: A descriptive qualitative study***

Date of interview…………………………………..

Interview site………………………...

Interviewee No ………………………

Start time……………………………

End time………………………………

**A: Interviewee background**

Age of participant----------------

Sex ------------------------------------------

Marital status---------------------------------------

Occupation -------------------------------------------

Level of education----------------------------------------

Years of working experiencet --------------------------------------------

Duration of caregiving………………………………………..

**B: Nurses’ experience**

1. Please describe your experiences communicating with patients’ relatives admitted in ICU.
2. Please share with me challenges that you faced when interacting with the relatives.
3. How did you navigate through the challenges.
4. What are the things that should be done to improve communication interaction.

Thank you for your time in this interview; do you have anything else to add? If not, I appreciate it very much!

**END**
