# Supplementary material for: Barriers to effective communication among nurses and family members of patients admitted to the intensive care unit at Muhimbili National Hospital in Dar es Salaam: A descriptive qualitative study
Source: PLoS One. 2025 Sep 4;20(9):e0330374. doi: 10.1371/journal.pone.0330374 (PMC12410765; doi:10.1371/journal.pone.0330374)
Supplement: S3 Text — (DOCX) [file pone.0330374.s003.docx]

**Barriers to effective communication among nurses and family members of patients admitted to intensive care unit at Muhimbili National Hospital in Dar es Salaam: A descriptive qualitative study**

**Table 1. Participants characteristics**

|  |  | **Family member s(n=12)** | **Nurses (n=15)** |
| --- | --- | --- | --- |
| Gender | Male | 3 | 6 |
|  | Female | 9 | 9 |
| Age | Below 30 | 0 | 0 |
|  | 30-49 | 11 | 15 |
|  | 50-69 | 1 | 0 |
| Level of education | Primary education | 5 | 0 |
|  | Secondary education | 2 | 0 |
|  | College and above | 5 | 15 |
| Years of working experience | Less than 5 years |  | 6 |
|  | More than 5 years |  | 9 |
| Duration of caregiving | Less than 2 weeks | 8 |  |
|  | More than two weeks | 4 |  |
| Relationship to the  patient admitted in ICU | Parent | 5 |  |
|  | spouse | 4 |  |
|  | Sibling | 1 |  |
|  | Others | 2 |  |
| Employment status | Formal employment | 4 |  |
|  | Self-employed | 8 |  |

**Excerpts of the transcript**

*“Sometimes, you want to ask a nurse for information about your patient, but instead, you’re asked, ‘Why do you want to know all that? Just see your patient and go.” (Relative 12)*

*“Some of us didn’t know hospital rules. I touched my patient without sanitizing, and the nurse shouted at me instead of explaining kindly.” (Relative 6)*

*“If I’m treated poorly, I go home wondering… what happens to my patient who can’t speak for himself?” (Relative 3)*

*“It’s easier to talk with educated family members; they understand you. With others, you have to simplify everything to get understood*.” (Nurse 8)

*“It happens that after sharing some information with a representative about their patients, when they go to share with the rest, they say something different…”* (Nurse 2)

*“When family members arrive at the hospital already convinced that nurses are harsh or indifferent to their concerns, it creates an immediate barrier to establishing trust and good cooperation.”(Nurse 4)*

*“Patients admitted to the ICU are very sick and need close observation. You become so occupied with the care to the point you don’t have time to talk much with patient family members.”* ( Nurse 7)

*“You may find yourself caring for two critically ill patients. In that kind of situation, it becomes difficult to have meaningful conversations with family members about their patients.”* ( Nurse 10)

*“We are allowed to visit our patients just for a while, that’s not even enough for nurses themselves to share information with us.” (Relative 2)*

*“We normally provide patient information to family members at the bedside. If it’s something very confidential, then we ask for space in the in-charge’s office for privacy or the doctor’s room.”* (Nurse 3*)*

*“You may want to ask the nurse sensitive information about your patient, but you find a lot of other family members with their patients… so you refrain… one time I asked the nurse, she spoke loudly… everyone around could hear...”* (Relative 9)

*“I can’t share everything, some details only doctors disclose. But families keep asking us, and we’re stuck saying, ‘Ask the doctor,’ which feels dismissive.”* (Nurse 1)

*“You ask the nurse about your patient, and you get a response like ‘go ask the doctor,’ but I always see the nurse with my patient. When you tell me to ask the doctor… where?” (Relative 4)*
