# Supplementary material for: Barriers to effective communication among nurses and family members of patients admitted to the intensive care unit at Muhimbili National Hospital in Dar es Salaam: A descriptive qualitative study
Source: PLoS One. 2025 Sep 4;20(9):e0330374. doi: 10.1371/journal.pone.0330374 (PMC12410765; doi:10.1371/journal.pone.0330374)
Supplement: Attachment — Submitted filename: Reviewers Comments.docx [file pone.0330374.s004.docx]

Thank you for the opportunity to review this paper titled *“Barriers to effective communication among nurses and family members of patients admitted to the intensive care unit at Muhimbili National Hospital in Dar es Salaam: A descriptive qualitative study.”* This paper has merits as it provides insights into improving communication between ICU nurses and family members of patients. The paper is generally well-written, and the following comments can improve this paper.

General comment

There many typographical errors that need attention. Authors should inspect all sentences

Abstract

- Well-written and clear

**Introduction**

- Line 48, correct the typographical error
- Facts are presented in line 49 to 52, but there is no citation provided
- The authors are reverting to the use of long-form and short form of the term ‘intensive care unit’, The principle of use of abbreviation needs to be followed for consistency

**Methods**

- There are many ICUs at MNH. However, information is lacking regarding their description in the study setting and which ICUs were included and why, and how the participants were selected across the ICUs.
- In line 103, ICU charge nurses should be revised, it is not clear
- There is a typo of full stop in line 110
- The sentence from line 114 to 116 needs revision
- Line 143 has a bracket typo

**Results**

- Male and female in line 152 should be in plural
- A sentence in line 152 - 153 needs to be revised to read “The majority, participants (n=26; 96.3%), were aged between 30 and 49 years”
- In line 157, information regarding the initial analysis that yielded 52 codes should be omitted. Authors should only present the results and not the process of analysis
- Authors should only present findings and avoid discussion. Statements such as in line 185 to 186 (These insights underscore how individual nurse behavior can deeply influence family members’ trust, emotional state, and perception of care quality) should be avoided in the findings section. They should be part of the discussion.

**Discussion**

- Citations in line 255 – 256 need to be merged
- Sentences from line 297 – 301 need to be revised for clarity
- Authors may expand on limitations to include social desirability

**Conclusion**

Well-summarized
