# Supplementary material for: Barriers to effective communication among nurses and family members of patients admitted to the intensive care unit at Muhimbili National Hospital in Dar es Salaam: A descriptive qualitative study
Source: PLoS One. 2025 Sep 4;20(9):e0330374. doi: 10.1371/journal.pone.0330374 (PMC12410765; doi:10.1371/journal.pone.0330374)
Supplement: Attachment — Submitted filename: Response to Reviewers.docx [file pone.0330374.s006.docx]

July 15^th^, 2025

To

Editor-in-Chief

PLOS ONE

Dear Sir/Madam,

On behalf of my co-authors, I am grateful for the valuable and constructive review of our manuscript entitled **“Barriers to effective communication among nurses and family members of patients admitted to the intensive care unit at Muhimbili National Hospital in Dar es Salaam: A descriptive qualitative study”**. We have revised the manuscript as per the reviewers’ comments and made clarifications where necessary. The specific response to reviewers’ comments is elaborated in detail within the matrix response sheet below, following this letter.

Yours sincerely,

Menti L. Ndile (*Corresponding Author)*

E-mail: [menti.ndile@muhas.ac.tz](mailto:menti.ndile@muhas.ac.tz)

|  | **Reviewer 1** | |
| --- | --- | --- |
|  | ***General comment*** There many typographical errors that need attention. Authors should inspect all sentences | Thank you for the observation. We have gone through the manuscript thoroughly to correct any typographical errors. We have also used a language editor for correction. |
|  | ***Abstract*** |  |
|  | • Well-written and clear | Thank you |
|  | ***Introduction*** |  |
|  | • Line 49, correct the typographical error | Corrected the sentence and replaced “Heathcare systems” to “healthcare systems” after the comma |
|  | • Facts are presented in line 49 to 52, but there is no citation provided | Thank you for the comment. The statement has been improved, and the citation (no 3) added. Line 49 to 51. |
|  | • The authors are reverting to the use of long-form and short form of the term ‘intensive care unit’, The principle of use of abbreviation needs to be followed for consistency | Thank you for the observation. We have revised the document and consistently applied the principle of the use of abbreviations. See lines 23,66 and 79 |
|  | ***Methods***  •There are many ICUs at MNH. However, information is lacking regarding their description in the study setting and which ICUs were included and why, and how the participants were selected across the ICUs. | Thank you for the comment. More information has been added to describe the type of ICUs involved, the reason, and the selection process. See lines  78 to 80 and 87 to 91. |
|  | • In line 103, ICU charge nurses should be revised, it is not clear | Thank you. The sentence has been rephrased to “Nurse managers from medical and surgical ICUs …” This improves clarity. Now in line 105. |
|  | • There is a typo of full stop in line 110 | Thank you for the observation. the type has been corrected. Currently line 113. |
|  | • The sentence from line 114 to 116 needs revision | Thank you. The sentence has been revised to read” The audio-recorded interviews were transcribed verbatim. An inductive thematic framework by Braun and Clarke’s six-step framework [9] was employed to identify patterns and themes directly from the data” This has brought more clarity than the previous sentence. Now in line 117 to 119 |
|  | • Line 143 has a bracket typo | Thank you, the error has been removed-Line 145 |
|  | ***Results*** |  |
|  | • Male and female in line 152 should be in plural | Thank you for the observation. The typo has been corrected. Line 157 |
|  | • A sentence in line 152 - 153 needs to be revised to read “The majority, participants (n=26; 96.3%), were aged between 30 and 49 years” | Thank you, the sentence has been revised as suggested. Line 157 to 158. |
|  | • In line 157, information regarding the initial analysis that yielded 52 codes should be omitted. Authors should only present the results and not the process of analysis | Thank you for the comment. The sentence about code generation has been removed, and the sentence has been revised-Line 162 to 164. |
|  | • Authors should only present findings and avoid discussion. Statements such as in line 185 to 186 (These insights underscore how individual nurse behavior can deeply influence family members’ trust, emotional state, and perception of care quality) should be avoided in the findings section. They should be part of the discussion. | Thank you for the comment. The Results section has been revised to present the findings objectively, with all interpretive or discursive statements removed. |
|  | ***Discussion*** • Citations in line 255 – 256 need to be merged | Thank you for the observation. The citations have been merged. Now in line 253. |
|  | • Sentences from line 297 – 301 need to be revised for clarity | Thank you. The sentences have been revised to improve clarity. Now in line 295 to 299 |
|  | • Authors may expand on limitations to include social desirability | Thank you for the suggestion. We have incorporated a sentence on social desirability bias. Now line 322-325. |
|  | ***Conclusion*** Well-summarized | Thank you |
|  | **Reviewer 2** | |
|  | 1. In astract, line no 21-22, as this is the report, I think it should read "This atudy aimed at..." | Thank you for the observation. This has been corrected. |
|  | 2. The backgroud is too long, I suppose you rephrase it. | Thank you for the suggestion; however, I have decided to retain the background as it is, as the word count is 310 (not too long) and is sufficient to contextualize the study. |
|  | 3. Line no 86, there is no need of citing (no idea what was cited here). If need be, the citation ahould be made in line 90-91(definition of saturation) | Thank you for the comment. The definition of purpose sampling is inserted corresponding to the citation. Now line 86-87. |
|  | 4. There is a bracket hanging in line 143 (a single bracket) | Thank you. This has been corrected. Now line 145. |
|  | 5. In table 1, since your sampling was purposive, why could'nt you balance the gender between participants? | Thank you for the observation. There was an attempt to have a representative sample; however, the nature of the study population is that most of the relatives visiting their patients and nurses working in the hospital are females. This has been added as a limitation of the study-line 325-327 |
|  | 6. Since there is no any participant below the age of 30, is it necessary for this category to be included? | Thank you for the comment. The age categories have been reorganized accordingly. Table 1 |
|  | 7. In line 254-255, I suppose all the four citation stay in the same bracket, e.g. [15-19] instead of [15, 16] [17-19] | Thank you for the observation. This has been corrected as suggested. Now in line 253. |
